# Supplementary material for: MetaRibo-Seq measures translation in microbiomes
Source: Nat Commun. 2020 Jun 29;11:3268. doi: 10.1038/s41467-020-17081-z (PMC7324362; doi:10.1038/s41467-020-17081-z)
Supplement: Supplementary file 10 — Supplementary Data 7 [file 41467_2020_17081_MOESM10_ESM.zip › File2/Confidence_High_Taxonomy/110242_out.krona.html]

Javascript must be enabled to view this page.

members
magnitude
magnitudeUnassigned
count
unassigned
taxon
rank

110242\_out

19

superkingdom
2
19

19
976
phylum

200643
class
19

19
order
171549

family
815
1

1
genus
816

1
species
1638780

SRS014948\_contig\_number\_22253

family
2005525
18

2
375288

SRS018133\_contig\_number\_6440SRS101268\_contig\_number\_909
18
genus

16
species
46503

SRS013951\_contig\_number\_24154SRS044535\_contig\_number\_contig-100\_27549.27550SRS044535\_contig\_number\_contig-100\_18255.96317SRS054905\_contig\_number\_contig-100\_25149.72247SRS063190\_contig\_number\_2576SRS063489\_contig\_number\_contig-100\_2037.61131SRS074670\_contig\_number\_2542SRS078419\_contig\_number\_contig-100\_2668.2668SRS104327\_contig\_number\_contig-100\_12859.12860SRS1055069\_contig\_number\_contig-100\_12189.12190SRS143148\_contig\_number\_contig-100\_5292.89720SRS144135\_contig\_number\_contig-100\_3032.203034SRS144506\_contig\_number\_contig-100\_7144.60263SRS148091\_contig\_number\_contig-100\_13324.13325SRS148721\_contig\_number\_contig-100\_25089.75468SRS148817\_contig\_number\_contig-100\_9032.70724
